# Plasma microRNA detection standardization test

**DOI:** 10.1002/jcla.23058

**Published:** 2019-10-15

**Authors:** Xiaomin Feng, Yuwei Liu, Nan Wan

**Affiliations:** ^1^ Dalian Medical University Dalian Liaoning China; ^2^ Department of Laboratory Medicine Science Center General Hospital of Northern Theater Command Shenyang Liaoning China

**Keywords:** microRNA, myocardial infarction, risk score, standardization

## Abstract

**Background:**

MicroRNAs (miRNAs) can be used for early diagnosis of myocardial infarction. However, due to a lack of standardized operating procedures, their value for clinical application is low.

**Methods:**

Detection of plasma miRNAs was optimized by analyzing factors influencing miRNA variance and myocardial infarction risk scores during analysis (extraction, reverse transcription, and real‐time PCR) and pre‐analysis (dietary status, anticoagulants, storage conditions, and hemolysis).

**Results:**

Regarding variable factors during analysis, the centrifugal column method was superior to Trizol LS reagent when extracting miRNA from plasma. Recovery rate was highest with plasma volumes of 200 and 300 µL. During analysis, the main source of miRNA detection inaccuracy was derived from RNA extraction (mainly organic extraction), and not reverse transcription or PCR. MiRNA variance could be reduced by use of an internal reference. During analysis, 95% of risk score variation fluctuated within a range of 6.267. The variable factors pre‐analysis mainly involved dietary status, anticoagulant selection, and storage conditions. Hemolysis positively correlated with miRNA levels, but there was no significant change in risk score after internal reference calibration.

**Conclusion:**

Preliminary standardization for miRNA detection provides a reference for clinical blood testing of miRNAs.

## 1 INTRODUCTION

Many studies have shown that microRNAs (miRNAs) can be used for early diagnosis and prognosis of myocardial infarction, but because the detection technology is still developing, it has been difficult to promote use of the approach in the clinic.^1^ Many variables cause variability in miRNA detection, and lack of systematic analysis of these variables exacerbates the problem. Indeed, clinical utility of miRNAs is still in the discovery and biological verification phase, with little technical verification performed, which also leads to uncertainty regarding repeatability of miRNA detection. MiRNAs are endogenous noncoding small‐molecule single‐stranded RNAs that regulate gene expression at the post‐transcriptional level.^2^ MiRNAs regulate growth and participate in many physiological and pathological processes, including cardiovascular disease.^3^ As miR‐126 and miR‐92a are widely present in endothelial cells (EC) and endothelial progenitor cells, their levels are significantly reduced in the plasma of patients with myocardial infarction.^4^ Consequently, we detected variation in miR‐126 and miR‐92a levels to identify factors affecting quantification of miRNA. U6 was used as an internal reference and cel‐miR‐39(cel‐39) as an external reference. In this study, we provide information on testing and improvements to optimize and standardize miRNA detection for clinical application.

## 2 MATERIALS AND METHODS

### 2.1 Study population

Twenty‐three healthy subjects and eight patients who underwent coronary angiography with myocardial infarction were selected for the study in September 2018. A diagnosis of myocardial infarction was based on the 2007 American College of Cardiology Foundation/American Heart Association (ACCF/AHA) guidelines.^5^ Exclusion criteria were as follows: severe liver and kidney disease, previous myocardial infarction or cerebral infarction, and malignant tumors. This study was conducted in accordance with the guidelines of the Helsinki Declaration and was approved by the Medical Ethics Committee of General Hospital of Shenyang Military Region (RER NO.[2018]40).

### 2.2 Detection of miRNA

Venous blood was collected from each patient into an EDTA‐K2 vacuum blood collection tube and then centrifuged at 4000 rpm (2862 × *g*) at 4°C for 15 minutes. Afterward, the plasma was separated into RNase‐free EP tubes and stored in a freezer at −80°C. Plasma miRNA was extracted using the EasyPure miRNA Kit (#ER601; TransGen Biotech Co., Ltd) or Trozol LS reagent(#10296010; Thermo Fisher Scientific Co., Ltd), in accordance with the manufacturer's instructions. All steps were performed on ice to avoid RNA degradation.

RNA reverse transcription was performed using the TransScript One‐Step gDNA Removal and cDNA Synthesis SuperMix kit (#AT311; TransGen Biotech Co., Ltd.), following the manufacturer's instructions. Real‐time fluorescence quantitative PCR was performed using the Light Cycler 480 SYBR Green Master (#04707516001; Roche) in a reaction volume of 20 μL including 5 μL reverse transcription product, 10 μL Master Mix, 1 μL forward primer, 1 μL reverse primer, and 3 μL nuclease‐free water. The reaction conditions were as follows: 95°C for 10 minutes, followed by 45 cycles of 95°C for 15 seconds and 60°C for 1 minute. Two replicates per sample were performed. Relative miR‐126 and miR‐92a expression was analyzed using the ΔCq method. All reactions were performed using a Roche 480 PCR Thermal Cycler. Each specimen was supplemented with 10 µL of 100 nmol/L *Caenorhabditis elegans* cel‐39 as an external reference. U6 was used as an internal reference.

### 2.3 Preparation of hemolyzed samples

Human plasma was divided into eight EP tubes. The plasma content of each tube was 1200 µL, and red blood cell lysate content was 6, 12, 15, 21, 30, 42, or 72 µL (Red Blood Cell Lysis Buffer, B541001; Sangon Biotech). Serially diluted hemolysis samples were prepared. The hemolysis index (HI) was calculated as follows: $\text{HI}=A_{414}-A_{385}+0.16\times A_{385}$ 6 (A_414_ and A_385_ reflect absorption peaks at 414 and 385 nm, respectively). Hemolysis was measured using a spectrophotometer (Youke Instrument) and hemocytometer (Coulter HL780; Beckman Coulter).

### 2.4 Preparation of aqueous phase samples

Purchased miR‐126, miR‐92a, and cel‐39 were combined at the same 1:1:1 ratio for eight identical aqueous samples. Four were organically extracted as group A. For the other four aqueous samples (group B), these were directly added to spin columns and eluted with RNase‐free water.

### 2.5 Myocardial risk score

The risk score was calculated as follows: $\text{RS}=\sum i\;w_{i}\;x_{i}+2.573$, where *w* represents the miRNA weighting coefficient and *x* the miRNA expression value (ΔCq) after calibration with the internal reference.

### 2.6 Efficiency of miRNA isolation for cel‐39

$$\text{cel}-39\;\text{recovery}=\frac{\text{cel}-39\;\text{addition}-\text{cel}-39\;\text{measurement}}{\text{cel}-39\;\text{addition}}\times 100\%$$

### 2.7 Statistical analysis

Data were analyzed using SPSS22.0 (IBM) and MedCalc 15.0 (MedCalc Software). Independent‐sample *t* test was used for normally distributed data, and chi‐square test was used for the comparison of rates. The cycle value (Cq) for miRNA detection is represented by a histogram, and variance is represented by a box plot. Risk scores are represented by normal distribution maps and line graphs. Spearman's correlation was used to examine correlation of HI with hemolysis percentage and hemoglobin concentration (mg/L), and correlation of hemolysis percentage with Cq value and risk miRNA score. Heat maps were generated using Microsoft Excel. Differences were considered statistically significant at *P* < .05.

## 3 RESULTS

### 3.1 Results of clinical characteristics and baseline demographics of patients in two groups

Table 1 showsa comparison of demographic and baseline data between the AMI group and the control group. There were no significant differences in TC and Scr between two groups. However, age, Male ratio, TG, Glu, ALT, and the past history ratio were significantly higher in the AMI group than the control group.

**Table 1:** Clinical characteristics and baseline demographics of patients

Characteristics	AMI group	Control group	*P*
N	8	23	
Demographics			
Male (n/%)	6/2 (75.0)	11 (47.8)	<.001
Age (y)	54.88 ± 16.80	29.47 ± 7.49	.003
Laboratory indicators			
TC (mmol/L)	4.56 ± 1.03	3.84 ± 0.60	.095
TG (mmol/L)	1.89 ± 1.29	0.92 ± 0.55	.006
Glu (mmol/L)	8.63 ± 2.44	5.22 ± 0.75	.005
ALT (U/L)	43.20 ± 26.44	17.64 ± 8.74	.029
Scr (μmol/L)	77.94 ± 24.82	65.12 ± 18.86	.138
Past history (n/%)			
Hypertension	1 (12.5)	1 (4.4)	<.001
Family history	1 (12.5)	0	<.001
Smoking	6 (75.0)	4 (17.4)	<.001

Note

The continuous variables are listed as mean ± standard deviation.

Abbreviations: ALT, Alanine aminotransferase; Glu, glucose; Scr, Serum creatinine; TC, Total cholesterol; TG, Triglyceride.

### 3.2 Variation in analysis: Extraction, reverse transcription, and real‐time PCR

#### 3.2.1 The effect of extraction kit type and plasma volume on extraction

**Figure 1:**
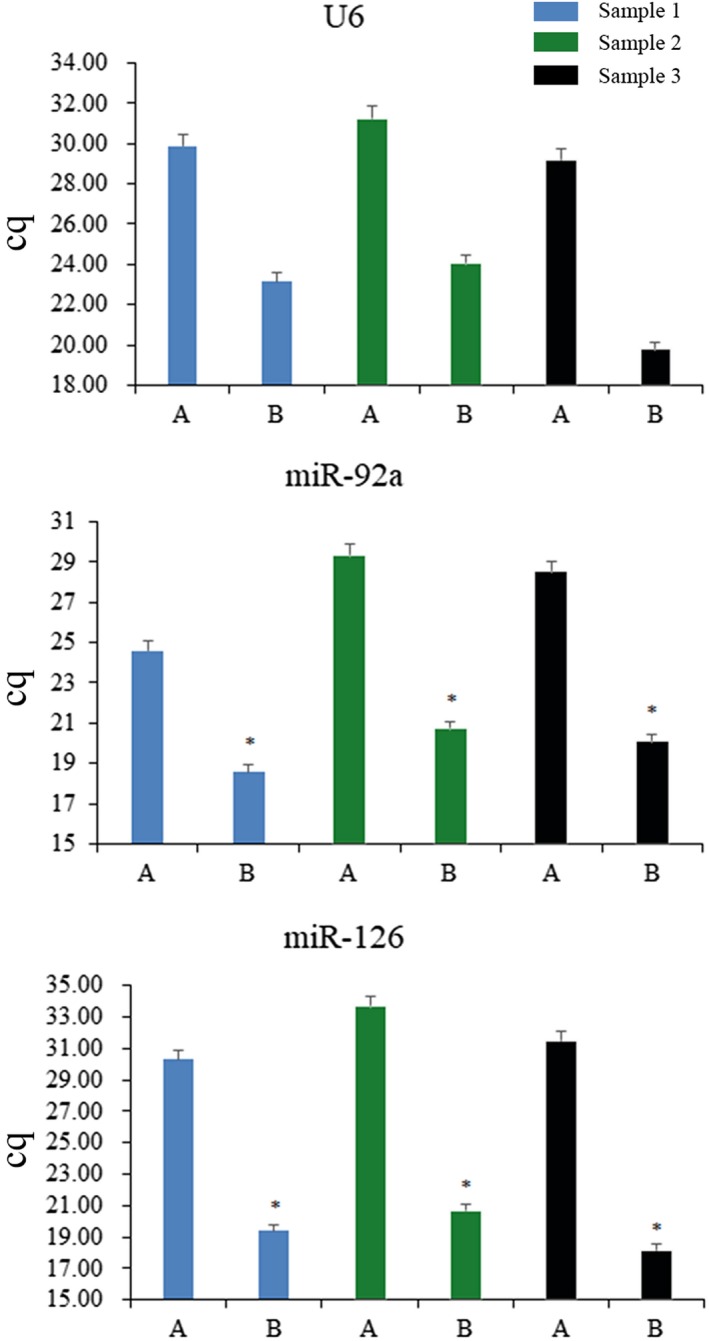
Effect of different extraction protocols on miRNA extraction. Group A was the extraction of miRNA from plasma using Trizol LS. And group B was extracted by centrifugal column method. Asterisks show statistically significant differences between groups A and B, *P* < .05

**Figure 2:**
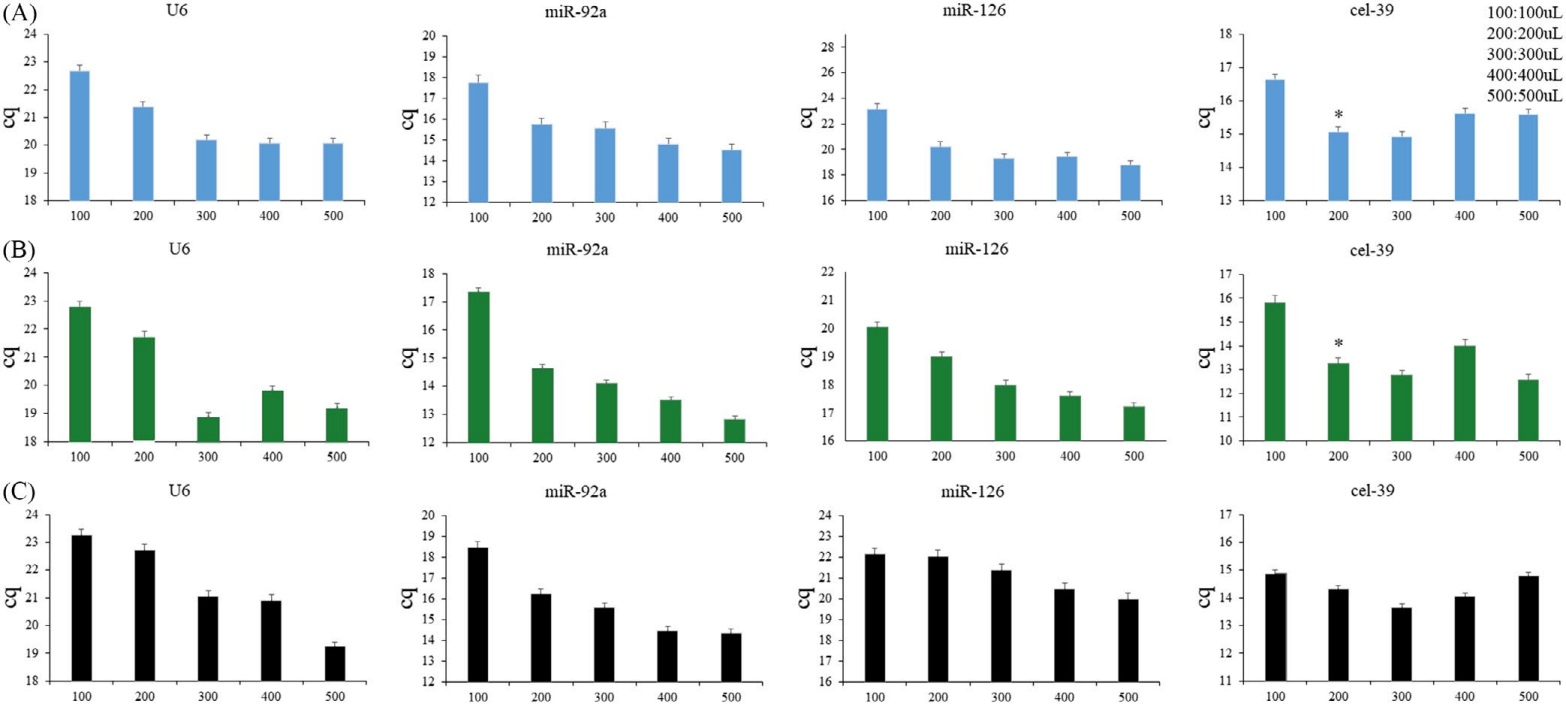
Comparison of the effect of different plasma starting volumes on miRNA extraction. MiR‐126, miR‐92a, U6, and cel‐39 in three samples: A, B, and C were quantified by quantitative PCR. Cel‐39 was used to monitor extraction efficiency (miRNA recovery rate). Asterisks show statistically significant differences between plasma volumes of 200 µL and 100, 400, or 500 µL, *P* < .05

Two kits were used to extract RNA: Trizol LS and ER601. The TransGen Biotech centrifugal column method showed better performance and resulted in a higher yield (ie, lower Cq value; Figure 1). Therefore, we chose the centrifugal column method for miRNA extraction. Initial plasma volumes of 100, 200, 300, 400, and 500 µL were selected for extraction. As plasma volume increased, Cq values gradually decreased. By measuring recovery of the external reference, we obtained highest recovery when the plasma volume was 200 or 300 µL (Figure 2). Further, recovery decreased with plasma volumes of 400 or 500 µL. Since recovery was highest at 200 or 300 µL, and the maximum volume that can be processed in a 1.5‐mL EP tube is 200 µL, we subsequently selected 200 µL plasma for miRNA extraction. Typically, 500 µL of aqueous phase is produced per 200 µL plasma. To prevent contamination of the organic phase, we collected 200 µL (40%) of aqueous phase for further processing, which resulted in a reduction of external reference recovery to 37.4% (as expected), but had no effect on reproducibility of the extraction process.

#### 3.2.2 Effect of extraction, reverse transcription, and real‐time PCR on miRNA detection

**Figure 3:**
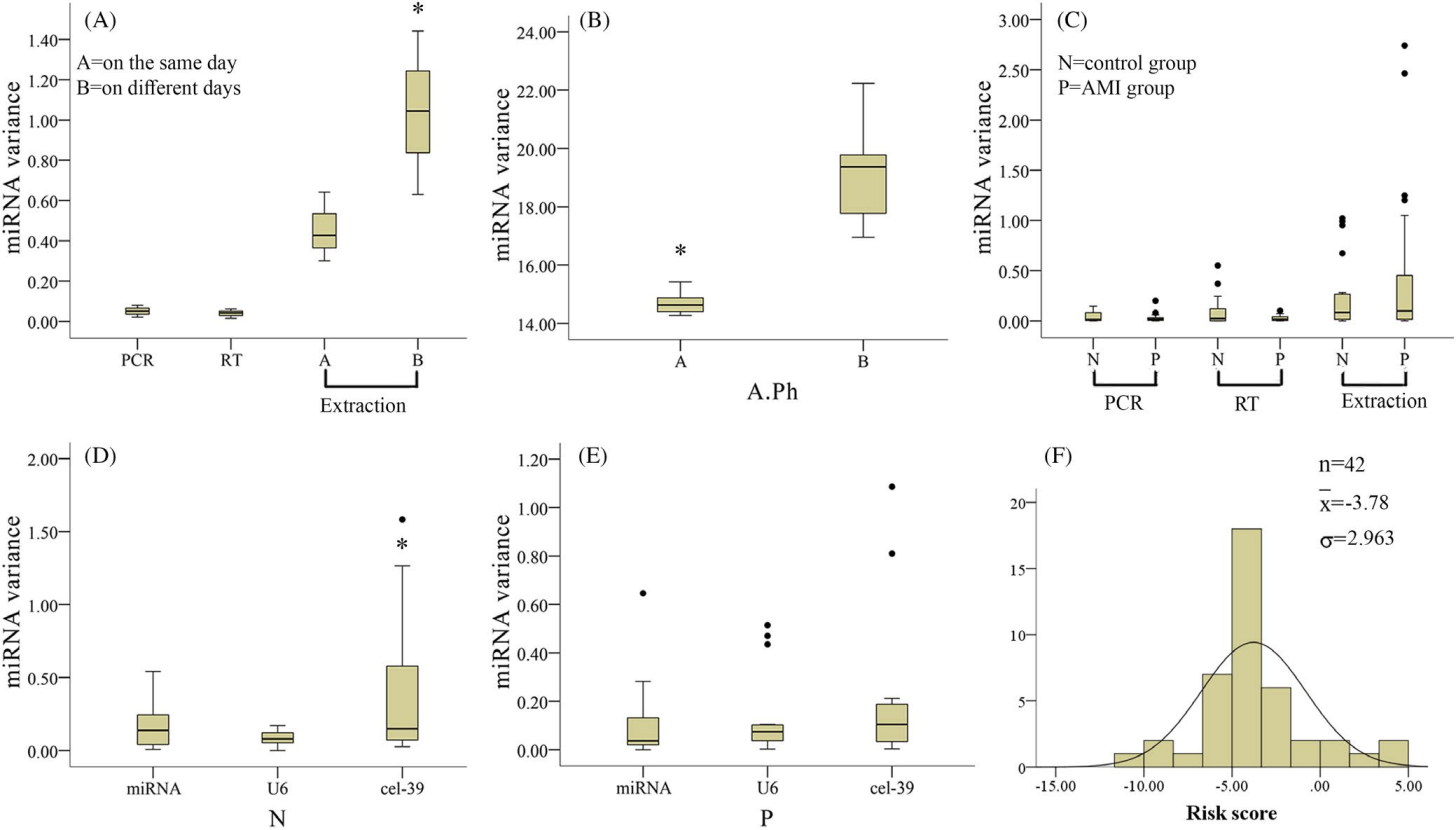
Optimization for detection of circulating miRNAs by extraction, reverse transcription, and PCR. A, Using the same plasma, multiple (n = 4) reverse transcription and PCR processes were performed to determine the effect of these processes on miRNA detection variability. Extraction was performed on the same day (group A) or different days (group B) (n = 4). B, Four aqueous phase samples were organically extracted (group A), while the other four aqueous samples were directly added into spin columns and eluted with RNase‐free water (group B). C, The effect of extraction, reverse transcription, and PCR on variability of miRNA detection in a control group and acute myocardial infarction (AMI) group. D and E, Influence of miRNA data before and after calibration on accuracy of measurements in the control group and AMI group. F, Calculation of risk scores in 54 previously analyzed plasma samples after internal reference calibration. In (A), the asterisk indicates a significant difference between group B and A, *P* < .05. In (B), the asterisk indicates a statistically significant difference between group A and group B, *P* < .05. In (D), an asterisk indicates a statistically significant difference between the cel‐39 group and miRNA and U6 groups, *P* < .05

Differences in miRNA detection were mainly concentrated in the extraction step rather than with reverse transcription or PCR (Figure 3A). PCR and reverse transcription had little effect on predictability of the test results (<0.2 Cq). There were significant differences between groups B and A (*P* < .05; Figure 3A). A total of eight identical aqueous phase samples were combined. The four aqueous phase samples of group A were subjected to organic extraction. All four aqueous samples of group B were directly added to the spin column and eluted with RNase‐free water. Group B eliminated differences caused by organic extraction. Moreover, the water phase group exhibited a decrease in variance (*P* < .05; Figure 3B), which corresponded to 93.9% variance of the entire procedure.

#### 3.2.3 Validation of a small number of clinical samples

Different clinical samples were used to examine variability of miRNA detection. Plasma of eight patients with myocardial infarction diagnosed by coronary angiography and eight healthy subjects was obtained. Variation between the reverse transcription and PCR groups was small, yet greater with the extraction group (>0.3 Cq; Figure 3C). When internal and external parameters were used to calibrate the PCR results, external parameters did not reduce variability between experiments, while internal parameters greatly reduced variability (<0.2 Cq; Figure 3D and E).

#### 3.2.4 Assessing the impact of measurement variability on risk scores

Risk scores of 42 previously tested plasma samples were calculated. Risk scores were normally distributed (Figure 3F) with a mean and standard deviation of −3.78 ± 3.138. Thus, during analysis, 95% of risk score variation fluctuated within a range of 5.926.

### 3.3 Variation pre‐analysis: Sample collection and storage

#### 3.3.1 Effect of dietary status on miRNA detection

**Figure 4:**
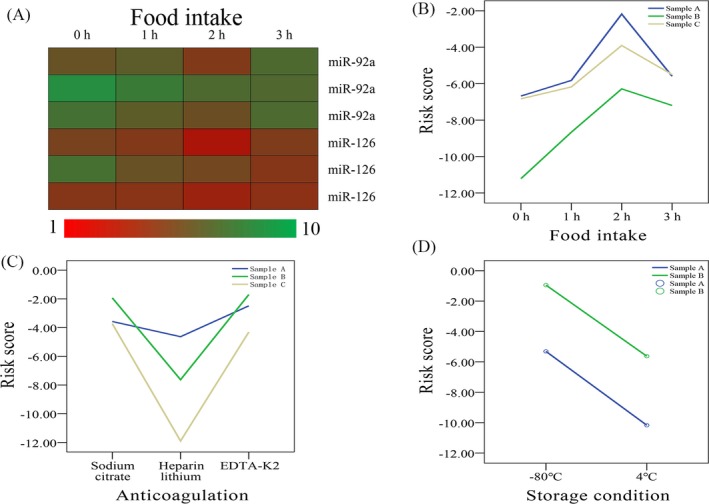
Effect of pre‐analysis variation on circulating miRNA levels and miR‐Test risk scores. A, Differences in circulating miRNA expression in three healthy individuals when blood was collected under different dietary conditions: fasting (0 h) or 1, 2, or 3 h after food intake. The rows represent ΔCq values of each miRNA in each individual after calibration with internal parameters (red for reduction; green for increase). B, Fluctuation in miR‐Test risk scores in different samples for each individual under different dietary conditions. C, Fluctuation in miR‐Test risk scores in different samples for each individual using different anticoagulants (sodium citrate, heparin lithium, or EDTA‐K2). D, Fluctuation in miR‐Test risk scores in different samples for each individual under different storage conditions (stored at −80°C or 4°C before analysis)

Venous blood was taken at fasting (0 hour) and 1, 2, and 3 hours after eating. There were statistically significant differences in plasma miRNA content before and after eating (*P* < .05, Figure 4A). Risk score after eating significantly increased, but began to decline after 3 hours (Figure 4B).

#### 3.3.2 Effect of different anticoagulants on miRNA detection

Anticoagulation was performed with sodium citrate, heparin lithium, and EDTA‐K2. miRNA expression and risk score were significantly reduced using heparin lithium anticoagulant (>35 Cq; data not shown). There were no significant differences in miRNA content and risk score between EDTA‐K2 and sodium citrate (*P* > .05, Figure 4C).

#### 3.3.3 Effect of storage conditions on miRNA detection

Storage conditions greatly affected miRNA levels and risk scores (Figure 4D). Compared with samples stored at −80°C immediately after plasma separation, the same individual plasma sample showed significant reductions in miRNA levels after storage for 1 week at 4°C, with a decrease of 2.76 ± 1.53 Cq. Significantly reduced risk scores were also found (Figure 4D).

#### 3.3.4 Effect of hemolysis on miRNA detection

**Figure 5:**
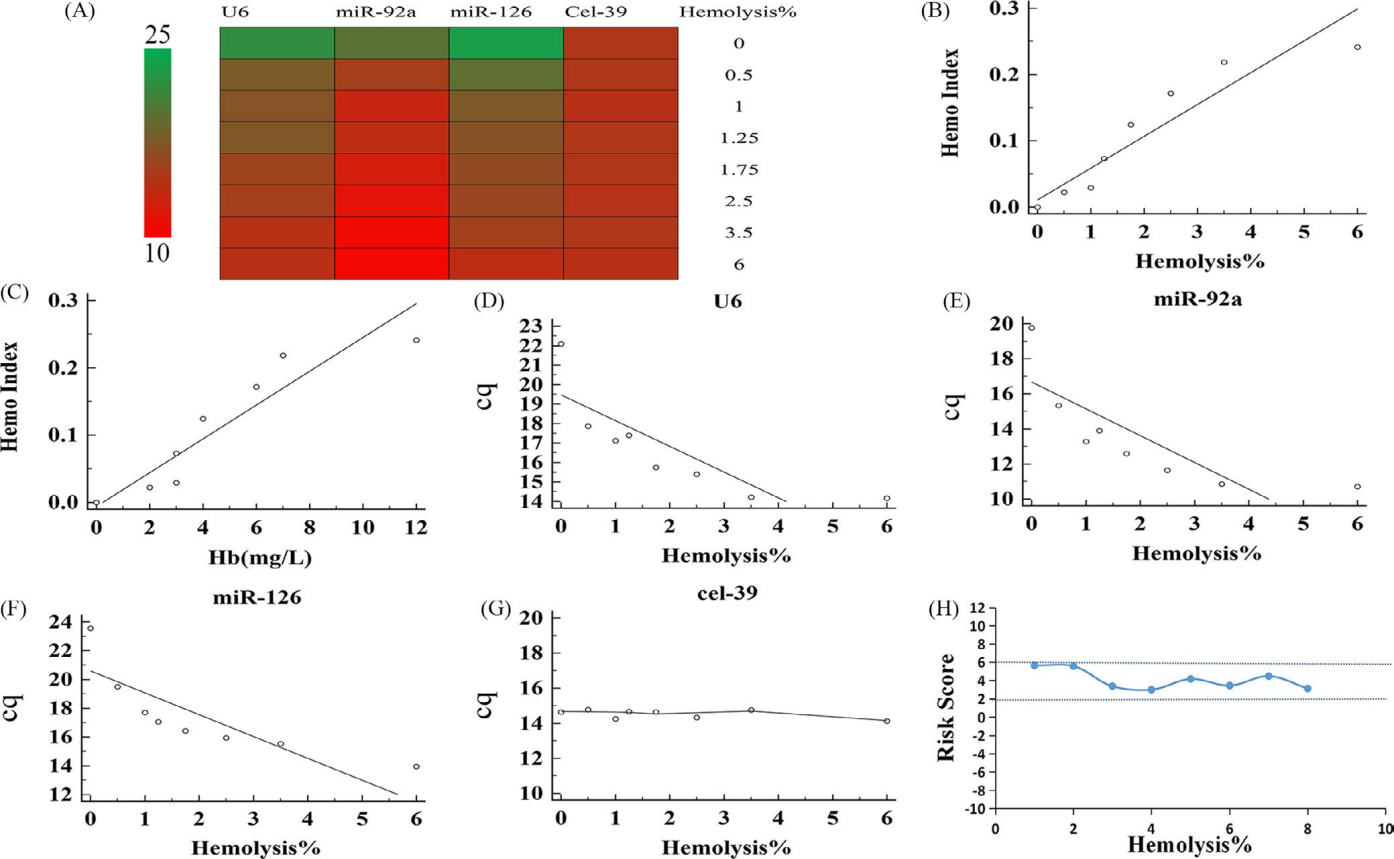
Effect of hemolysis on circulating miRNA quantification and risk scores. A, Differences in miRNA expression in plasma samples with different hemolysis percentages (red represents reduced; green represents increased). B and C, Spearman's correlation was used to examine correlation between the hemolysis index and hemolysis percentage and hemoglobin concentration (mg/L). D‐G, Spearman's correlation was used to examine correlation between hemolysis percentage and miRNA Cq values. H, Effect of hemolysis percentage on miRNA risk scores

Hemolysis resulted in increases in miR‐126, miR‐92a, and U6 levels, while cel‐39 showed no change (Figure 5A). Hemolysis percentage and hemoglobin concentration were highly correlated with HI (*r*_1_ = 0.93, *P*_1_ < 0.01; *r*_2_ = 0.99, *P*_2_ < 0.01, respectively; Figure 5B and C). Without calibration, miRNA levels were directly proportional to the degree of hemolysis, except for cel‐39 (*r*_U6_ = −0.8, *P*_U6_ = 0.02; *r*_92a_ = −0.78, *P*_92a_ = 0.02; *r*_126_ = −0.75, *P*_126_ = 0.03; *r*_39_ = −0.53, *P*_39_ = 0.17; Figure 4D‐G). However, after calibration with an internal reference, risk score did not change significantly due to the degree of hemolysis (Figure 4H), indicating that the internal reference eliminated the changes caused by hemolysis.

## 4 DISCUSSION

This study examined nonspecific fluctuations of current miRNA detection systems using variables of miRNA detection performance and reproducibility.

When analyzing variables, the RNA extraction step was the main source of inaccuracy, not reverse transcription or PCR.^7^ Variability of the extraction process may be due to differences in total miRNA recovery, with highest recovery at a plasma volume of 200 or 300 µL. Recovery did not increase with increasing plasma volume and in fact decreased at plasma volumes of 400 and 500 µL. A possible reason for this is that when the amount of plasma is too large, the extraction step is cumbersome and some steps may need to be repeated, which increases loss of miRNA and thus reduces recovery rate. Alternatively, this could have occurred because the increase in plasma volume introduces excessive contaminants (highly variable plasma matrix, lipid, or plasma protein),^6^ which interferes with the purification process, as often occurs in clinical samples. Won reported that in the absence of a reliable method of miRNA isolation from plasma,^8^ selection of an appropriate internal or external parameter can reduce variation in results due to detection factors.^9^ In this experiment, we introduced internal and external references to calibrate the results and found that using internal parameters reduces this variation considerably. In contrast, variation increased when using external parameters, which may be due to instability of the external parameters. We suggest that the external reference should be diluted and quantified to observe stability of the external reference. Further, an appropriate external reference should be selected for each specific experiment, although we currently believe that use of an endogenous reference is better.^10^

Pre‐analysis variability often affects miRNA levels. Accordingly, it has been reported that specimen hemolysis can cause plasma contamination, leading to increased miRNA levels.^11^ Studies have found that although miRNA levels increase during hemolysis, because some miRNAs in red blood cells are present at high levels,^12^ they can be calibrated by an internal reference and risk scores are not affected. Therefore, we believe that miRNA detection is not affected by hemolysis. However, dietary status before blood collection, storage status after specimen collection, and choice of anticoagulant greatly affects risk score, which may interfere with a clinician's judgment on the patient's condition. Changes in risk score after eating may be due to lipid interference in plasma. High triglyceride levels inhibit Taq DNA polymerase activity and affect miRNA amplification efficiency.^13^ Thus, we suggest that blood collection should be performed under strict fasting conditions. Plasma should be stored immediately in a freezer at −80°C, with miRNA content determined as soon as possible after extraction to minimize miRNA degradation. When collecting blood samples, use of lithium heparin anticoagulation tubes should be avoided, because heparin inhibits the activity of Taq DNA polymerase to affect the quantification of miRNA.^14^ Consequently, it is recommended to strictly follow the operating procedures when collecting and preparing samples. Additionally, establish complete sampling (blood collection and separation of plasma), standard procedures for detection (RNA extraction, reverse transcription, and PCR), and analysis to reduce and discover nonspecific fluctuations in the detection process.

At present, we need a large number of clinical samples to verify and evaluate every noted concern. Our current findings show that data calibration can eliminate most errors. Nonetheless, these nonspecific issues still affect accuracy of miRNA detection. The greatest concerns still need to be overcome, for example correct selection of internal and external parameters and stability of internal parameters in the sample.

In summary, we have described optimization and standardization of cyclic miRNA detection for clinical application. To ensure accuracy of results when changing from laboratory research to clinical diagnosis, the above optimization criteria are important factors that must be considered.

## CONFLICT OF INTEREST

The authors declare that they have no conflict of interest.

## AUTHOR CONTRIBUTIONS

All authors declare that they were involved in this work and are fully responsible for the findings.
